# A review of the risk factors, genetics and treatment of endometriosis in Chinese women: a comparative update

**DOI:** 10.1186/s12978-018-0506-7

**Published:** 2018-05-21

**Authors:** Yi Dai, Xiaoyan Li, Jinghua Shi, Jinhua Leng

**Affiliations:** Department of Obstetrics and Gynecology, Peking Union Medical College Hospital, 1# Shuaifuyuan, Dongcheng District, Beijing, 100730 China

**Keywords:** Endometriosis, Chinese, Risk factors, Mutations, Treatment

## Abstract

Endometriosis is one of the major causes of economic burden and compromised quality of life in a very large percentage of Asian women. While it is perceived as a benign condition, recent research has shown that it may be a significant cause of infertility and metastatic cancer. It has also been associated with other diseases linked to the functioning of the immune system. Genetic as well as environmental factors are known to affect the manifestation and progression of endometriosis. This review aims to summarize recent research pertaining to the risk factors, diagnosis and treatment of endometriosis in Chinese women. It also provides an overview of identified genetic mutations and polymorphisms and their effects on the risk of developing endometriosis in the Chinese population. A comparison has been drawn between Asian and European-American female populations and the differences in risk factors and treatment responses have been summarized. Since traditional Chinese medicine (TCM) is often used to treat endometriosis, wherever possible, a comparison between efficacies of Western medicine and TCM in the Chinese population has also been provided. Although much progress has been made in the treatment and resolution of endometriosis, several gaps remain and this review also highlights possible areas of future research and advancement that can result in an improvement in patient outcomes and quality of life.

## Plain English summary

Endometriosis is a disorder in which the stromal or glandular tissue that normally lines the inside of the uterus grows in a location outside of the uterus. About 10–15% of women in their reproductive years are affected worldwide. Although the cause of endometriosis remains unclear, genetic and environmental factors are considered as risk factors for manifestation and progression of endometriosis. This study aims to summarize recent research pertaining to the risk factors, diagnosis and treatment of endometriosis in Chinese women. Women with endometriosis generally suffer from severe pain and other debilitating consequences which results in a compromised quality of life. Endometriosis also has a major effect on the child-bearing ability of women. Studies show that though several strategies for the management of affected patients have been developed, complete cure is not yet possible. Endometriosis treatment generally involves medications or surgery. Traditional Chinese medicine (TCM) that is often used for infertility treatment has shown successful results in controlling the recurrence of endometriosis following surgery thereby providing symptomatic pain relief and improving Health Related Quality of Life. Though several treatment regimens are available for the management of patients with endometrial lesions, alternative strategies are used in China. In conclusion, although Western medicine has been studied and validated more extensively for the treatment of endometriosis, both TCM and Western medicine are used equally in the treatment of endometriosis in Chinese women.

## Background

Endometriosis is an estrogen-responsive, chronic condition that arises from the extra-uterine growth of the stromal or glandular tissue that lines the uterus [1]. Worldwide, it represents a significant cause of morbidity in about 10-15% of women in their reproductive years [2]. Although significant efforts have been made to enhance detection and subsequently treat endometriosis, diagnosis in the majority of women is delayed on average by 10 years globally [3] and by 13 years in China [4]. Identification of risk factors is integral to diagnose endometriosis. Moreover, early diagnosis is essential to effectively treat endometriosis. This review delineates the risk factors and genetic differences among Chinese women suffering from endometriosis and also updates the reader about the treatment of endometriosis in China.

### Endometriosis: Pathophysiology and risk factors

The risk of endometriosis has been linked to ethnicity and several studies have reported a nine-fold increase in risk in Asian women when compared to the European-American white female population [5–7].

It is well known that endometriosis, despite having a strong genetic predisposition, is also affected by environmental exposures. Both these factors have a bearing on the difference in the risk of disease occurrence observed based on parameters such as race and ethnicity. While evidence for an association between genetic polymorphisms and risk of endometriosis is robust [8–11], evidence linking environmental factors to endometriosis risk is somewhat weaker. Environmental factors such as elevated levels of phthalate esters, persistent organochlorine pollutants, perfluorochemicals, and intra-uterine exposure to cigarette smoke among others can cause endometriosis by inducing oxidative stress, altering hormonal homeostasis, or by changing immune responses. However, further studies in different populations are required in order to measure the extent of this association [12–16]. Other risk factors, such as the presence of lower genital tract infections, have also been proposed as a possible cause of increased risk [17].

**Fig. 1:**
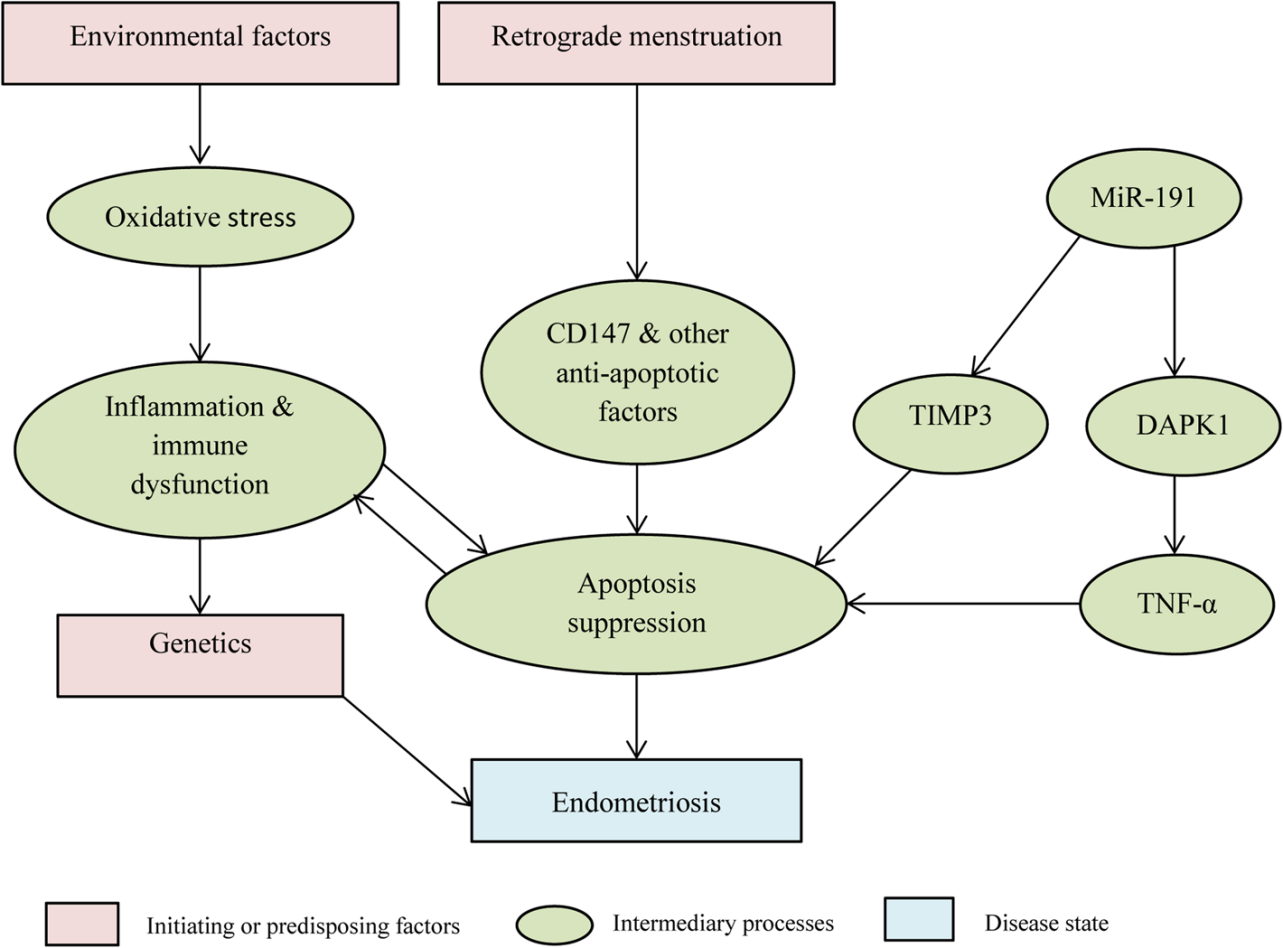
Overview of the proposed interplay between various factors reported in the pathogenesis of endometriosis. The different factors are denoted by the various shapes and the arrows indicate the interplay between them. Abbreviations: CD147 – Basigin or cluster of differentiation 147, MiR-191 – Micro RNA 191, TIMP3 – Tissue Inhibitor of Metalloproteinase 3, DAPK1 – Death-associated protein kinase 1, TNF-α – Tumor Necrosis Factor α

The origin and pathophysiology of endometriosis remains incompletely understood and several hypotheses that seek to explain its development and progression have been proposed (Fig. 1). The most well accepted model is that of retrograde menstruation (where endometrial cells are refluxed through the fallopian tubes and implanted onto the pelvic or peritoneal organs) accompanied by the avoidance of anoikis, wherein cells of endometrial origin able to survive outside the uterus have an imbalance between pro and anti-apoptotic factors [18, 19]. It has been reported that CD147 is one such anti-apoptotic factor and its overexpression has been shown to lead to the survival of human uterine epithelial cells outside the uterus [20]. The miRNA MiR-191 has also been shown to be involved and inhibits tumor necrosis factor-α induced apoptosis of ovarian endometriosis and endometrioid carcinoma cells by targeting Death-associated Protein Kinase 1 [21]. Certain molecules such as fibroblast growth factor receptor 1 have also been shown to be overexpressed in ectopic versus eutopic endometrium and in patients suffering from endometriosis versus healthy controls. One study also demonstrated an overexpression of fibroblast growth factor receptor 1 in patients with post-surgery recurrence [22]. In addition, studies have also revealed the involvement of miRNAs and siRNAs in disease pathology. In fact, the process of endometriosis associated ovarian cancer is thought to involve miR-191. By down-regulating and lowering protein expression of tissue inhibitor of metalloproteinases 3, which has a pro-apoptotic function, it is thought to induce the survival of ectopic endometrial cells. Interestingly, lower tissue inhibitor of metalloproteinases 3 expression has also been associated with several other types of cancer [23].

Other theories have also been put forward to explain the origin of endometriosis. Non-uterine theories propose the occurrence of coleomic metaplasia, a transdifferentiation of extrauterine tissue to ectopic endometrial tissue, probably due to endocrine disrupting chemicals or other hormonal factors [24]. A recently proposed theory suggests that bone marrow-derived stem cells can cause endometriosis. This occurs due to increased binding of the stem cell chemokine CXCR4 with its ligand CXCL12, which increases matrix metalloproteinase expression and subsequent extracellular matrix protein degradation, leading to metastasis. Furthermore, CXCR4-CXCL12 binding promotes angiogenesis through increased vascular endothelial growth factor expression [25].

Symptomatically, endometriosis is characterized by inflammation and involvement of different immune system components. Lesions may be divided into three main types, viz., peritoneal, ovarian, and deep infiltrating endometriosis, depending on their location within the body. Several pelvic and peritoneal organs may be involved, and although the involvement of the urinary tract and kidneys are rare, a single study has reported the presence of endometriosis of the renal parenchyma in a Chinese woman [26].

In general, a large degree of variation in disease severity exists amongst patients who suffer from this condition. It has also been found that an increase in the number of endometrial lesions does not always correlate with the severity of symptoms, suggesting that endometriosis is a multifaceted condition that is governed by several aspects of patient physiology. Based on global standards, disease is classified as Stage I-IV based on revised criteria pertaining to extent and severity specified by the American Society of Reproductive Medicine (rASRM), also known as the revised American Fertility Society criteria. However, this method was not robust enough to predict post-surgery pregnancy outcome [27]. To counter rASRM’s shortcoming, a new staging system called the Endometriosis Fertility Index (EFI) was developed. EFI evaluated age, duration of infertility, pregnancy history, extent of endometriosis, and the least-function score to predict pregnancy outcome after surgery [28]. The Chinese Medical Association (CMA) recommends utilizing EFI to assess outcomes in endometriosis patients [29].

Women with endometriosis generally suffer from severe pain and other debilitating consequences such as dysmenorrhea, chronic pelvic pain, dyspareunia, and ex-genital symptoms such as dyschezia, dysuria, hematuria, and rectal bleeding resulting in a compromised quality of life (QoL) [30, 31]. Another major effect of harboring this condition is infertility and 30–50% of the women affected by endometriosis have compromised child-bearing ability [32]. A recent systematic review analyzing the outcomes of more than 1.9 million women found endometriosis to be associated with worsening of obstetric and pregnancy related outcomes such as miscarriage, pre-term labor, placenta previa, small for gestational age, and cesarean delivery as compared with healthy controls [33]. A Chinese study also reported similar results [34]. Apart from these consequences, there is also an increased risk of diseases such as cancer (especially ovarian) [35–37] and auto-immune diseases. Studies have also shown that tumors from Chinese ovarian cancer patients with pre-existing endometriosis have distinct clinico-pathological features, such as a predisposition for ovarian clear cell carcinoma, when compared to the normal Chinese population [38].

In general, the consequences of endometriosis are often under-reported and underestimated [39] and the disease represents a significant burden, both economic and qualitative [40]. Although several strategies for the management of affected patients have been developed, complete cure is not yet possible. Even after treatment with current therapeutic regimens including surgical intervention, endometriosis remains a frequently recurring disease. The following sections of this review seek to summarize the state of the art in the diagnosis and treatment of this disease, and wherever possible, to draw a comparison between strategies used in China with those deployed in the Western world for the effective resolution of disease and management of patients.

### Genetic basis of endometriosis in Chinese women

Mutations and polymorphisms in several genes have been implicated in the pathophysiological process that results in endometriosis. These genes belong to diverse groups, both functionally as well as spatially. However, a strong association has been detected between single nucleotide polymorphisms (SNPs) in hormone receptor and metabolism related genes and endometriosis in Taiwanese Chinese Women [41]. A study in Taiwanese women also showed that MUC17 polymorphisms are involved in endometriosis development and associated infertility [11]. In addition, by comparing the mutational landscape between normal women and Chinese endometriosis patients, a recent whole exome sequencing study implicated genes involved in biological adhesion, cell-cell junctions, and chromatin-remodeling complexes in the development of endometriosis [42].

Much research based on genetic factors that influence endometriosis has focused on Han Chinese women, since a relatively higher rate of disease prevalence has been detected in this population. In addition, genome wide association studies found that polymorphisms in rs12700667 located within the intergenic region of 7p15.2 are also associated with an elevated risk of ovarian endometriosis in North Chinese women [43]. Although several studies have detected differences in genetic mutations that predispose Asian and Western populations to endometriosis, a recent genome wide association meta-analysis revealed a significant overlap in loci linked with endometriosis risk in Japanese and European populations [44].

Mutation association studies conducted in different populations in order to link genetic mutations to risk of endometriosis presents a very strong body of evidence supported by a large number of meta-analyses. Some of these associations related to Chinese and Asian populations have been summarized in Table 1.

**Table 1:** Genetic Mutations and Polymorphisms and their impact on the risk of Endometriosis and Infertility in Chinese women

Gene/ Protein Name	Protein Function	Mutation/ Polymorphism	Risk of Endometriosis/Infertility	Population	References
ESR 1 Estrogen receptor alpha	Hormone Receptor	(TA)n Short	Increased/Un-reported	Mixed	Wang et al., 2013 [86]
		(TA)n Long	Decreased/Unreported	Mixed	Wang et al., 2013 [86]
		rs3798573 A/G	Increased/Increased	Han Chinese	Wang et al., 2013 [86]
ESR 2 Estrogen receptor beta	Hormone Receptor	rs4986938 and rs1256049 polymorphisms	No significant association detected/ Unreported	Asian and European-American	Guo et al., 2014 [87]
PR Progesterone Receptor	Hormone Receptor	rs104283 CT SNP	Increased/Un-reported	Southern Han Chinese	Mao et al., 2015 [88]
GST Glutathione-S-transferases M1/T1	Metabolic Enzyme	Null genotype	Increased/ Unreported	Chinese	Chen et al., 2015 [9]; Zhu et al., 2014 [89]
GALT Galactose-1-phosphate uridyl transferase	Metabolic Enzyme	Q188R and N314D	No significant association detected/ Unreported	Chinese	He et al., 2006 [90]
BDNF Human brain-derived neurotrophic factor	Tropic factor	Val66Met polymorphism	Increased/Increased	Han Chinese	Zhang et al.,2012 [91]
FGF2 Fibroblast Growth Factor 2	Growth factor	754C/G polymorphism	Increased/Un-reported	North Chinese	Kang et al., 2012 [92]
VEGF Vascular endothelial growth factor	Growth factor	+405G > C	No significant association detected/ Unreported	Asian and European-American	Fang et al., 2015 [93]
		-1154A	Decreased/Unreported	North Chinese	Liu et al., 2009 [94]
		-2578A	Decreased/Unreported	North Chinese	Liu et al., 2009 [94]
VEGFR-2 Vascular endothelial growth factor receptor 2)	Growth factor receptor	1192C/T + T/T	Decreased	Han Chinese	Kang et al., 2013 [95]
		1192C/C	Increased		
TP53	Tumor suppressor	codon 72 polymorphism Pro/Pro and Arg/Pro	Increased	Chinese and Asian	Chang et al., 2002 [96]; Jia et al., 2012 [97]
MMP-2 Matrix metalloproteinase-2		1306C-- > T and -735C-- > T	Increased/Unreported	North Chinese	Kang et al., 2008 [98]
TIMP-2 Tissue inhibitor of metalloproteinase-2		418G-- > C	Decreased/Unreported	North Chinese	Kang et al., 2008 [98]
E-Cadherin	Cell Adhesion Molecule	rs8049282 SNP	Increased/Increased	Northern Chinese	Kang et al., 2014 [99]
COX-2 Cyclo-oxygenase 2	Inflammatory pathway Enzyme	G to A at − 1195 (promoter)	Increased/ Unreported	Chinese	Wang et al., 2015 [100]
ICAM-1 Intercellular Adhesion Molecule 1	Cell Adhesion Molecule	K469E polymorphism	Further decreased in Asian populations compared to European-Americans/Unreported	Asian and European-American	Pabalan et al., 2015 [101]
CYP19	Aromatase Enzyme	rs700518AA	No significant association detected/Increased upon pre-existing endometriosis	Chinese	Wang et al., 2014 [102]
FCRL3		rs7528684	Unreported/ Increased upon pre-existing endometriosis	Han Chinese	Zhang et al., 2015 [103]
IL-16 Interleukin 16	Cytokine	rs4778889 T/C polymorphism	Increased/ Unreported	Chinese	Gan et al., 2010 [104]
FSHR Follicle Stimulating Hormone Receptor	Hormone receptor	SNP: 680Ser/Ser and 680Ser/Asn	Decreased/Unreported	Taiwanese Chinese	Wang et al., 2011 [105]
XRCC4 X-ray repair cross-complementing group 4	DNA repair gene	codon 247*A	Increased/Unreported	Taiwanese Chinese	Hsieh et al.,2008 [106]
		promoter-1394*T	Increased/Unreported		
		Intron 3 I/D polymorphism	No significant association detected/ Unreported		
FOXP3	Transcription factor	rs2280883	No significant association detected/Unreported	Han Chinese	Wu et al., 2013 [107]
		rs3761548			
		rs3761549			

### Current techniques for diagnosis and biomarkers of endometriosis

Imaging techniques such as color Doppler ultrasounds and CT/ MRI scans are recommended for the initial diagnosis of endometriosis by the CMA, although MRI primarily visualizes ovarian and not peritoneal endometriosis [39]. However, due to the necessity for a histological verification of the presence of endometrial glands/stroma combined with a laparoscopy (which is regarded as the current gold standard for the confirmation of the presence of endometrial lesions) and also due to several cases of misdiagnosis, an accurate identification of endometriosis occurs after an average of 6 years following initial onset [29, 45]. In conjunction with a laparoscopic diagnosis of endometriosis, a scoring system is generally used for the assessment of severity of disease. The most commonly used one is the revised American Fertility Society scoring system for the extent and severity of ectopic endometrial adhesions. The CMA guidelines also use the Endometriosis Fertility Index (EFI) scoring system in order to assess patient fertility related parameters.

The technique of laparoscopy, however, has several drawbacks of which its invasive nature and reliance on the skill of the surgeon for an accurate visual inspection of the pelvic cavity representing the major issues. In addition, it is not always capable of detecting deep infiltrating lesions, resulting in several undiagnosed cases. However, efforts are being made in order to devise non-invasive methods for the diagnosis of endometriosis and certain studies have demonstrated an association between elevated serum levels of CA125 in endometriosis patients, suggesting that they may be used as biomarkers during the diagnostic process in both, Asian as well as European-American populations [46]. Another study has also demonstrated the superiority of CA125 over the platelet-lymphocyte ratio in the diagnosis of moderate to severe endometriosis in Chinese women [47]. However, CA125 levels cannot be used as a diagnostic biomarker in isolation due to a low sensitivity and specificity for endometrial lesions. One of the major concerns of the use of CA-125 as a biomarker is that elevated serum levels have been detected in other gynecological pathologies as well. However, CMA guidelines indicate that CA-125 elevation may be useful for the detection of advanced stage endometriosis, endometriosis combined with adenomyosis or obvious pelvic inflammation, and also in the diagnosis of endometrioma rupture [48, 49].

Several other strategies are being developed and panels that detect levels of inflammatory and non-inflammatory markers are still not very specific for endometrial lesions [50, 51]. Amongst inflammatory markers a systematic review identified IL-8 as the best studied, with MCP-1 and CCL5 coming a close second [50]. Another strategy for non-invasive diagnosis of disease is the use of miRNA panels. A prospective study has also shown that the urine of affected patients had a distinct peptide pattern that can be developed into an assay for diagnosis [52].

Despite these advances, recent Cochrane reviews have concluded that none of the currently available techniques, whether lab-developed or already existing commercial platforms, are suitable for use as a replacement for laparoscopy or even as a diagnostic triage test [53, 54]. Thus, more emphasis on targeting endometriosis-specific markers for diagnostic purposes is required in order to develop a non-invasive diagnostic testing platform for accurate and non-invasive identification of disease.

### Current strategies for the management of endometriosis in China and worldwide

**Fig. 2:**
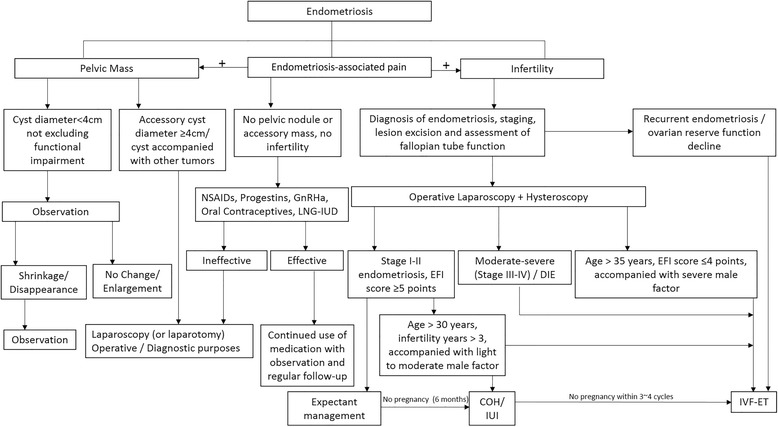
Diagnostic and treatment process for endometriosis patients in China. (EFI: Endometriosis Fertility Index). Adapted from CMA guidelines, 2015

Since endometriosis is associated with debilitating pain and a very high risk of infertility, most treatments aim to alleviate symptoms of the disease such as dysmenorrhea and dyspareunia while simultaneously improving pregnancy and fertility outcomes. Thus, the use of combinatorial regimens is common, and often, surgical excision or ablation of lesions using laparoscopy is often done in order to reduce large ectopic endometrial masses. However, based on recommendations by international guidelines, decisions regarding the timing and aptness of surgical intervention are generally guided by patient preferences, disease severity and fertility goals. Surgical removal is generally followed by GnRHa or oral contraceptives in order to prevent disease recurrence and to provide symptomatic relief. Clinical guidelines published by the Obstetrics and Gynecology branch of the CMA recommend specific treatment strategies based on presenting symptoms (i.e., only pain or pain with infertility) [29]. A flowchart outlining the diagnostic and treatment process for these two different types of patient populations in China are shown in Fig. 2 [29].

The following sub-sections of this review focus on the different types of treatment recommended for endometriosis and their advantages and drawbacks.

#### Surgical treatment

The goal of surgical treatment for endometriosis is to enable lesion resection, alleviate symptoms and improve fertility outcomes while simultaneously preserving internal anatomy. The decision of which type of surgery to undertake depends on the extent of endometriosis related pain and the fertility goals of the patient. Different types of surgical interventions can be used for the treatment of endometrial pain. However, such treatment is only recommended after the diagnosis of endometriosis has been histologically confirmed (after performing a laparoscopic examination).

A large body of clinical evidence supports the use of surgical laparoscopy to remove extraneous endometrial lesions. While guidelines recommend the use of ablation and surgical excision for the treatment of endometrial pain, such treatment is not recommended for advanced forms of the disease. Clinical guidelines also separate the type of surgical treatment to be used depending on whether ovarian endometriosis is detected. In general, the European Society of Human Reproduction and Embryology (ESHRE) guidelines recommend the use of cystectomy over drainage and coagulation and CO_2_ ablation in women with ovarian endometrioma [55]. The CMA specifically recommends laparoscopic surgery for cases in which endometriosis is accompanied by infertility and where large ovarian cysts (> 4 cm) are detected. In case of deep endometrial lesions, the CMA as well as ESHRE guidelines recommend surgical resection, with a caveat on the high complication rates arising from such treatment. In women who do not have fertility goals and fail to respond to conservative treatments, a hysterectomy is recommended by both sets of guidelines. In addition, surgical resection of nerve pathways by pre-sacral neurectomy is only recommended as an add on to conservative treatment, although this requires a very high degree of skill [29, 55].

#### Medical treatment

Several types of therapies have been recommended for the treatment of endometriosis by different clinical guidelines (depending on the presenting symptoms of the patient and the nature and extent of lesions), such as Non-Steroidal Anti-Inflammatory Drugs, oral contraceptives, progestins and GnRH-agonists. Guidelines also recommend the empirical treatment of endometriosis based on presenting symptoms only, without the need for a laparoscopic confirmation of diagnosis. In this regard, both the ESHRE and rASRM guidelines do not distinguish between first and second line treatment and recommend the use of NSAIDS, progestins (such as dienogest and danzol), oral contraceptives and GnRH agonists. The World Endometriosis Society (WES) guidelines recommend the use of NSAIDs, continuous oral contraceptives and progestins as first line treatment while GnRH agonists and the levonorgestrel intrauterine system (LNG-IUS) system are recommended as second line treatment. CMA guidelines indicate the use of empirical treatment in those cases where there is no obvious pelvic mass or infertility and first and second line treatment in these cases is aligned with the recommendations by the WES. The use of second line treatment is recommended after first line treatment has proved ineffective. In addition, the CMA recommends that surgery should be considered when second line treatment also fails. Effectiveness of each line of treatment can be checked using standard diagnostic procedures mentioned earlier.

While the treatment duration of NSAIDS has not been explicitly mentioned, the CMA recommends using oral contraceptives for 6 months or longer, progestins for 6 months, and GnRH agonists for 3 to 6 months or longer [29]. However, both the ESHRE and CMA recommend the continuous use of hormonal treatment post-surgery for the prevention of recurrence and previous studies have also shown that the discontinuation of hormonal therapy post-surgery leads to a higher rate of recurrence [56]. Specifically, the rASRM recommends the use of aromatase inhibitors, danazol, the LNG-IUS and medroxyprogesterone acetate as post-operative medical treatment. After a laparoscopic confirmation has been established, NSAIDs are recommended as the first line of treatment if lesions are minor and not of the deep infiltrating type.

In case of major symptoms and large, deep infiltrating lesions, hormonal therapy is recommended for use in combination with surgical excision by both, the CMA and ESHRE guidelines [29, 45]. Hormonal therapy may be divided into two categories, based on molecular targets. Broadly, they are classified as those that affect estrogen metabolism and responsiveness and those that affect progesterone responsiveness. A recent systematic review and meta-analysis conducted to assess the efficacy of the use of oral contraceptives after surgical excision of endometrial lesions concluded that combinatorial treatment is more effective in preventing recurrence than surgery alone. However, from their analysis the authors reported that this advantage did not extend to the improvement of pregnancy outcomes [57]. In a randomized, post-laparoscopic study of 280 Chinese women with ovarian endometriosis, the authors compared side effects and menopausal symptoms of leuprorelin and triptorelin (both GnRH antagonists) treatment. They reported that leuprorelin was milder than triptorelin, with a gradual reduction in hormone levels and fewer menopausal symptoms [58]. In another study of Chinese endometriosis patients that evaluated the timing of GnRH agonist administration (goserelin, 3.6 mg either 3–5 days post-operatively or on days 1–5 of menstruation) the authors reported that although the efficacy of treatment was equal in women with stage III-IV endometriosis, uterine bleeding over the course of the 28 day menstrual cycle was reduced in the former group [59]. A new crosslinked hyaluronan gel was also evaluated for its ability to reduce postoperative adhesions in a randomized study comprising of 215 Chinese women who underwent surgical laparoscopy for the initial removal of existing endometrial lesions. The study found that use of the gel decreased the number and severity of post-operative adnexal and abdominopelvic adhesions in patients, thus potentially reducing the incidence of disease recurrence [60]. In this study, the authors propose that since hyaluron has a sufficiently long elimination half -life (metabolic clearance is slowed down by its ability to cross-link), it is able to persist within the body for the time window during which new adhesions are formed.

Since genetic factors linked to ethnicity are known to affect the risk of developing endometriosis, studies have attempted to address the question of whether genetic differences extend to differences in responses to treatment. One such meta-analysis evaluated responses to GnRH agonists and to the progestin dienogest in European versus Japanese populations. Although the authors reported no differences in response rates or HRQoL parameters in either population and found both lines of treatment equally efficacious, dienogest treatment was found to be superior in terms of bone mineral density in both populations [61]. Some clinical evidence for genetically influenced racial disparity in endometrial cancer comes from a retrospective study which reported that African-American women experienced lower recurrence-free survival after using estrogen replacement therapy, possibly due to differences in estrogen metabolism [62]. However, further studies are required to understand the clinical implications of recently discovered racial differences, such as in microRNAs [63] and oncogene mutations [64].

Several alternative strategies have been used in order to improve the quality of life in women undergoing hormonal treatment for endometriosis and to reduce side effects of the therapy itself. For example, techniques such as progressive muscular relaxation training have been used effectively in order to reduce anxiety and depression in Chinese Han women receiving GnRH treatment for endometriosis [65].

In general, guidelines by the CMA, ESHRE and the rASRM encourage treatment strategies which aim to reduce and eliminate pain, recurrence and the use of multiple surgeries.

### Traditional Chinese medicine (TCM)

TCM is used in Chinese patients to control the recurrence of endometriosis following surgery, provide symptomatic pain relief, and improve Health Related Quality of Life (HRQoL). It is also often used to treat infertility. Some low-quality evidence from a Cochrane review showed Chinese herbal medicine to be superior to danazol treatment for the alleviation of symptoms such as pain and dysmenorrhea [66]. TCM users were also less likely to require surgical treatment for endometriosis than non-users [67]. Moreover, TCM was as effective as Western medicine (WM) in controlling the recurrence of pelvic endometriosis and improving fertility outcomes after conservative surgery [68], but better than WM at improving HRQoL [69]. On the other hand, a single randomized control trial demonstrated that TCM, and oral contraceptives in combination with laparoscopy were both non-superior to laparoscopy alone in the treatment of endometriosis [70]. Additionally, the underlying mechanisms of TCM remain unstudied. Furthermore, a major gap in knowledge is represented by the fact that Chinese medicine has never been compared with a placebo in the treatment of endometriosis symptoms, and hence, further research is required in order to confirm its effectiveness [66]. Thus, although Chinese medicine is often used for the management of endometriosis patients, there is a lack of high quality clinical evidence that supports its effectiveness in comparison with other mainstream treatment strategies.

Due to the absence of rigorous clinical evidence that supports the use of Traditional Chinese medicine for the treatment of endometriosis, the Delphi process (which is used in order to synthesize expert opinion for alternative medical interventions) was used in order to develop guidelines that govern the use of Chinese herbal medicine in the management of endometriosis patients [71]. This guideline informs practitioners about the different Chinese herbs that are used most commonly for the treatment of specific symptoms and outlines different patient management strategies based on an initial assessment of traditional Chinese physiology and pathology.

Thus, although several treatments exist for the management of patients with endometrial lesions, alternative strategies are commonly used in China. Since such strategies have not been adequately evaluated, further research that addresses this issue is required in order to improve patient outcomes, both in terms of fertility as well as quality of life.

#### Treatment of infertility

Since approximately 30%–50% of women suffering from endometriosis are infertile [32], its treatment constitutes a large part of the disease management plan. Previous work has established that the effectiveness of fertility treatment is inversely proportional to the severity of disease and that fertility outcomes in response to treatment are better in women with milder forms of endometriosis [72, 73]. This is probably due to poor ovarian reserve and oocyte quality and lower rates of implantation in women with stage III-IV endometriosis. Thus, the use of expectant management is an option only for women with less severe forms of endometriosis, and even then, guidelines recommend the use of controlled ovarian stimulation along with intra-uterine insemination for the improvement of fertility outcomes. However, those with infertility and advanced stage disease must receive effective treatment in order to improve fecundity. In general, medically assisted reproduction techniques such as ovulation induction and stimulation, intra-uterine insemination and other assisted reproduction techniques such as in-vitro fertilization (IVF) are recommended by most international guidelines including the CMA for the treatment of endometriosis associated infertility. In general, IVF is known to be highly effective in such cases and a meta-analysis by Barnhart et al., showed that the presence of endometriosis affected the fertility outcomes in patients receiving IVF only in cases of severe disease [74].

While medical treatments such as NSAIDS, oral contraceptives, progestins, and GnRH analogues are useful for the management of endometrial symptoms such as dyspareunia and dysmenorrhea, recent literature provides no evidence of their effectiveness for treating endometriosis related infertility in patients who desire a live birth. However, both GnRHa and oral contraceptives have been shown to improve outcomes in patients using IVF and assisted reproduction techniques [75].

Surgical treatment of endometriosis is an option for cases where mild to moderate disease is present as well as in cases where severe disease is detected along with poor fertility outcomes. However, the goal of surgery in these cases is to limit the extent of ovarian resection. Cumulative evidence has shown that laparoscopic surgery (by excision as well as ablation) is highly effective and significantly improves fertility outcomes in patients with minimal to moderate endometriosis already using Medically Assisted Reproduction (MAR) [76]. However, although evidence for its use in combination with other MAR techniques in severe cases is lacking; an individualized decision based on specific patient characteristics is recommended by all guidelines.

Fertility outcomes using combinatorial therapy were also evaluated in a retrospective study of 138 Chinese women. The authors concluded that GnRH agonists combined with the transvaginal ultrasound-guided cyst aspiration procedure results in improved therapeutic effects and pregnancy outcomes in infertile patients with ovarian endometriosis who underwent IVF-ET [77]. This represents a significant advancement, since the use of transvaginal ultrasound-guided cyst aspiration alone has been found to result in a very high rate of disease recurrence [78]. However, since these results are from a single study, current guidelines do not recommend this combination for the improvement of pregnancy outcomes. Another study in a group of 168 Chinese women with Stage III-IV endometriosis demonstrated that 2-month treatment with a GnRH agonist prior to IVF tended to increase the implantation rate, showing that the timing and duration of GnRH agonist therapy can also affect fertility outcomes [79]. However, current guidelines only recommend the use of GnRH agonists for the improvement of fertility outcomes in patients with severe disease (stage III-IV endometriosis based on rASRM classification) when used in combination with other surgical or MAR procedures [55].

It is believed that endometriosis occurs due to blood stasis which has manifested due to kidney Yang deficiency, liver Qi stagnation, or cold [80]. By targeting blood stasis, TCM aims to treat endometriosis and increase fertility. Indeed, a recent meta-analysis which compared TCM with WM found the former to improve pregnancy rates by almost 2-fold within 3–6 months of treatment initiation [80]. This mechanism treats endometriosis-related symptoms too. In endometriosis affected patients, the reduction in pain and adnexal mass with TCM was more than with danazol, but comparable with gestrinone. Compared with WM, a significant increase in pregnancy rate with TCM after laparoscopy (61.3% vs 45.5%, *P* < 0.05) [81], but not without (52.5% vs 37.5%, *P* = 0.265) was reported [82]. However, both the studies reported that TCM increases negative conversion of endometrial antibody significantly.

### Evaluation of HRQoL and clinical guidelines for the management of Chinese women with endometriosis

Since women with endometriosis are known to suffer from a significant deterioration in quality of life and fertility outcomes, HRQoL assessment is important during the clinical evaluation of treatment efficacy in endometriosis patients. Although generic Visual Analog Scales such as Short-Form 36 or Short-Form 12 and EuroQoL may be used to evaluate the efficacy of treatments in terms of HRQoL, several specific questionnaires that focus on different endometriosis-related QoL parameters such as self-image, relationship with children, effect on work life, and productivity have been developed. For example, the Endometriosis Health Profile-30 questionnaire which has been translated into Chinese [83, 84] and the much shorter Endometriosis Health Profile-5 questionnaire are commonly used for such assessments. In a study of 336 Chinese women, the authors found that the translated Endometriosis Health Profile-30 questionnaire was internally consistent and valid for use as an effective scale for the assessment of HRQoL in Chinese women [83].

Several international societies and organizations exist that regularly publish guidelines and updates for the management of endometriosis in patients. Among these, guidelines by the International Society for Gynecologic Endoscopy, the American Association of Gynecologic Laparoscopists, the European Society for Gynecological Endoscopy and the Australian Gynecological Endoscopy and Surgical Society are widely consulted. The ESHRE guidelines represent standardized clinical guidelines for the management of endometriosis worldwide [45, 55]. In general, the recommendations of the CMA are in line with international guidelines and discuss and propose different patient management strategies, as summarized in the above sections of this article.

Recently, the WES derived a set of consensus guidelines for the management of patients with endometriosis. This consensus statement was drafted after consultation with an international panel of experts and is the first guideline that represents the views of women affected by endometriosis themselves [85]. Of particular interest is the fact that these guidelines address the issue of patient management in under-studied groups such as adolescents and post-menopausal women. They also propose models for the management of disease in low resource settings, which is a significant concern in large and developing economies like China.

## Conclusions

In conclusion, although WM has been studied and validated more extensively for the treatment of endometriosis, TCM and WM are both used equally in the treatment of endometriosis in Chinese women. In addition, although guidelines recommend the use of different disease management strategies based on the extent and severity of endometrial lesions, long-term medical management is highly recommended in order to prevent exacerbation and recurrence. Guidelines by the CMA are aligned with those by the WES and ESHRE and in general, medical management is recommended over surgical treatment. More research is required on non-invasive diagnostic methods in order to accurately detect the presence of disease before a laparoscopy and to enable individualized treatment of patients while improving patient outcomes. While extensive studies have been conducted on the genetic basis of endometriosis in the female Chinese population, a clear association between the presence of mutations and polymorphisms and the clinical manifestation of disease has not been made. In addition, data regarding the underlying mechanisms involved are also limited and an extensive study of these factors is likely to allow clinicians to better manage the affected population.

## Abbreviations

CMA: Chinese Medical Association
EFI: Endometriosis Fertility Index
ESHRE: European Society of Human Reproduction and Embryology
GnRHa: Gonadotropin-releasing hormone agonist
HRQoL: Health Related Quality of Life
IVF: In-vitro fertilization
LNG-IUS: Levonorgestrel intrauterine system
MAR: Medically assisted reproduction
QOL: Quality of life
rASRM: American Society of Reproductive Medicine
SNP: Single nucleotide polymorphisms
TCM: Traditional Chinese medicine
WES: World Endometriosis Society
WM: Western Medicine
